# Predicting indirect effects of rotavirus vaccination programs on rotavirus mortality among children in 112 countries

**DOI:** 10.1038/s41541-023-00632-y

**Published:** 2023-03-04

**Authors:** A. N. M. Kraay, D. M. Chaney, A. Deshpande, V. E. Pitzer, B. A. Lopman

**Affiliations:** 1 Department of Kinesiology and Community Health, University of Illinois at Urbana-Champaign, Champaign, IL USA; 2 Institute for Genomic Biology, University of Illinois at Urbana-Champaign, Champaign, IL USA; 3 Hubert Department of Global Health, Rollins School of Public Health, Emory University, Atlanta, GA USA; 4 Department of Epidemiology, Rollins School of Public Health, Emory University, Atlanta, GA USA; 5 Department of Epidemiology of Microbial Diseases, Yale School of Public Health, Yale University, New Haven, CT USA

**Keywords:** Viral infection, Gastrointestinal diseases

## Abstract

Rotavirus is a leading cause of diarrhea deaths in children, particularly in low-to-middle income countries (LMICs). Licensed rotavirus vaccines provide strong direct protection, but their indirect effect—the protection due to reduced transmission—is not fully understood. We aimed to quantify the population-level effects of rotavirus vaccination and identify factors that drive indirect protection. We used an SIR-like transmission model to estimate the indirect effects of vaccination on rotavirus deaths in 112 LMICs. We performed a regression analysis to identify predictors of indirect effect magnitude (linear regression) and the occurrence of negative indirect effects (logistic regression). Indirect effects contributed to vaccine impacts in all regions, with effect sizes 8-years post-vaccine introduction ranging from 16.9% in the WHO European region to 1.0% in the Western Pacific region. Indirect effect estimates were higher in countries with higher under-5 mortality, higher vaccine coverage, and lower birth rates. Of the 112 countries analyzed, 18 (16%) had at least 1 year with a predicted negative indirect effect. Negative indirect effects were more common in countries with higher birth rate, lower under-5 mortality and lower vaccine coverage. Rotavirus vaccination may have a larger impact than would be expected from direct effects alone, but the impact of these indirect effects is expected to vary by country.

## Introduction

Rotavirus is a leading cause of diarrheal disease that accounts for 29.3% of diarrhea-related deaths in children under 5 years old^1^ and over 90% of these deaths occur in low-to-middle income countries (LMICs)^2^. Most children in LMICs will have multiple rotavirus infections by 2 years of age^3^. For these reasons, the burden of rotavirus is unequally placed on those living in LMICs.

There are four rotavirus vaccines (Rotarix, RotaTeq, Rotasiil, and Rotavac) that are licensed and recommended by the World Health Organization (WHO)^4,5^. Rotarix is the primary vaccine used in LMICs, as it has been on the market for far longer than Rotasiil and Rotavac; it requires two doses, typically administered around two and 4 months of age^6^. All four vaccines reduce the risk of rotavirus gastroenteritis in vaccinated individuals, known as the direct effect of the vaccine^4,7^. However, the strength of the direct effect varies by country. A study of the effectiveness of rotavirus vaccines in countries with different income-levels found the vaccine was ~84–90% effective in high-income countries compared with 75% in middle-income countries and 50% in low-income countries^7^.

The indirect effect of a vaccine is the protection provided to individuals in a partially vaccinated population as a result of the vaccine reducing transmission^8^, and is less well understood^9^. Biological and demographic factors can influence the magnitude of indirect effects^10^. Because the direct effect of rotavirus vaccination is smaller in LMICs, it is expected that LMICs will also gain less indirect protection from the rotavirus vaccine, but data on indirect impacts in these settings is limited^11^. One recent transmission modeling study estimated that indirect effects did enhance the overall impacts of rotavirus vaccination, but their impact varied by site^12^. More comprehensive analysis is needed to assess whether such indirect effects can be achieved consistently and to identify factors that might increase their impact.

As rotavirus vaccination expands, improving understanding of potential impacts, including indirect benefits, is important to optimize immunization policy. In this study, we utilize a previously published transmission model^12^ to predict the indirect effects of rotavirus vaccines from time of vaccine introduction to 12-years post-introduction in 112 LMICs (see Supplementary notes for full country list). We then use regression analysis to identify country-level predictors of modeled indirect effect magnitude.

## Results

### Estimation of vaccine effects by WHO geographic region

**Fig. 1:**
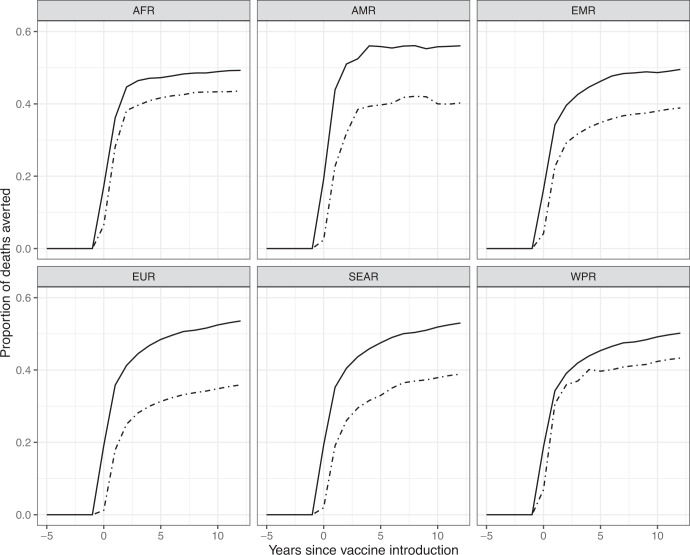
Annual median direct and indirect effect by WHO geographic region. Annual median population direct effect (dashed line) and overall effect (solid line) up to 12 years post vaccine introduction by WHO geographic region.

Overall, the transmission model predicted a 47.8% to 56.1% reduction in rotavirus deaths at the regional level at 8-years post-vaccine introduction, i.e., the overall effect (Table 1, Fig. 1). The Region of the Americas (AMR) had the highest median overall effect (56.1%; country range: 42.6%-61.7%), followed by Europe (EUR) (51.0%; country range: 47.0%-61.2%), Southeast Asia (SEAR) (50.4%; country range: 43.9%-55.9%), the Eastern Mediterranean (EMR) (48.6%; country range: 38.3%-62.1%), Africa (AFR) (48.5%; country range: 38.2%-59.4%), and the Western Pacific (WPR) (47.8%; country range: 4.4%-60.6%). While median relative reductions were highest in AMR, the median number of deaths averted across modeled countries was greatest in AFR due to the higher baseline burden of severe rotavirus in that region (Supplementary Fig. 1). The direct effect of vaccination was similar in all sites (regional average ranging from 33.7–43.2%). However, the proportion of overall benefit from rotavirus vaccination due to the indirect effect of vaccination (calculated as the indirect effect divided by the overall effect) varied widely amongst regions. In EUR, 33.1% of the median overall effect was due to the median indirect effect of the vaccine, followed by 28.3% in SEAR, 26.4% in AMR, 20.6% in EMR, 10.3% in AFR, and 2.0% in WPR.

**Table 1:** Median percent of rotavirus deaths averted 8-years post-vaccine introduction to due vaccine effects.

Region	Overall effect (%) (Country-level range)	Population direct effect (%) (Country-level range)	Indirect effect (%) (Country-level range)	Proportion of overall effect due to indirect Effect (IE/OE) (%) (Country-level range)
AFR	48.5 (38.2–59.4)	43.2 (33.5–54.7)	5.0 (−3.1–19.4)	10.8 (−6.4–33.5)
AMR	56.1 (42.6–61.7)	42.1 (28.0–51.8)	14.8 (2.1–26.7)	27.2 (3.9–45.1)
EMR	48.6 (38.3–62.1)	37.2 (30.6–52.2)	10.0 (−3.5–17.3)	21.0 (−8.5–29.6)
EUR	51.0 (47.0–61.2)	33.7 (27.1–50.2)	16.9 (2.9–23.9)	33.1 (5.9–46.8)
SEAR	50.4 (43.9–55.9)	36.9 (27.5–41.6)	14.3 (6.6–20.6)	27.1 (13.9–39.8)
WPR	47.8 (4.4–60.6)	41.2 (3.9–60.2)	0.97 (−1.7–21.2)	5.1 (−3.5–41.9)

Country-level values are shown in Supplementary Table 2.

**Fig. 2:**
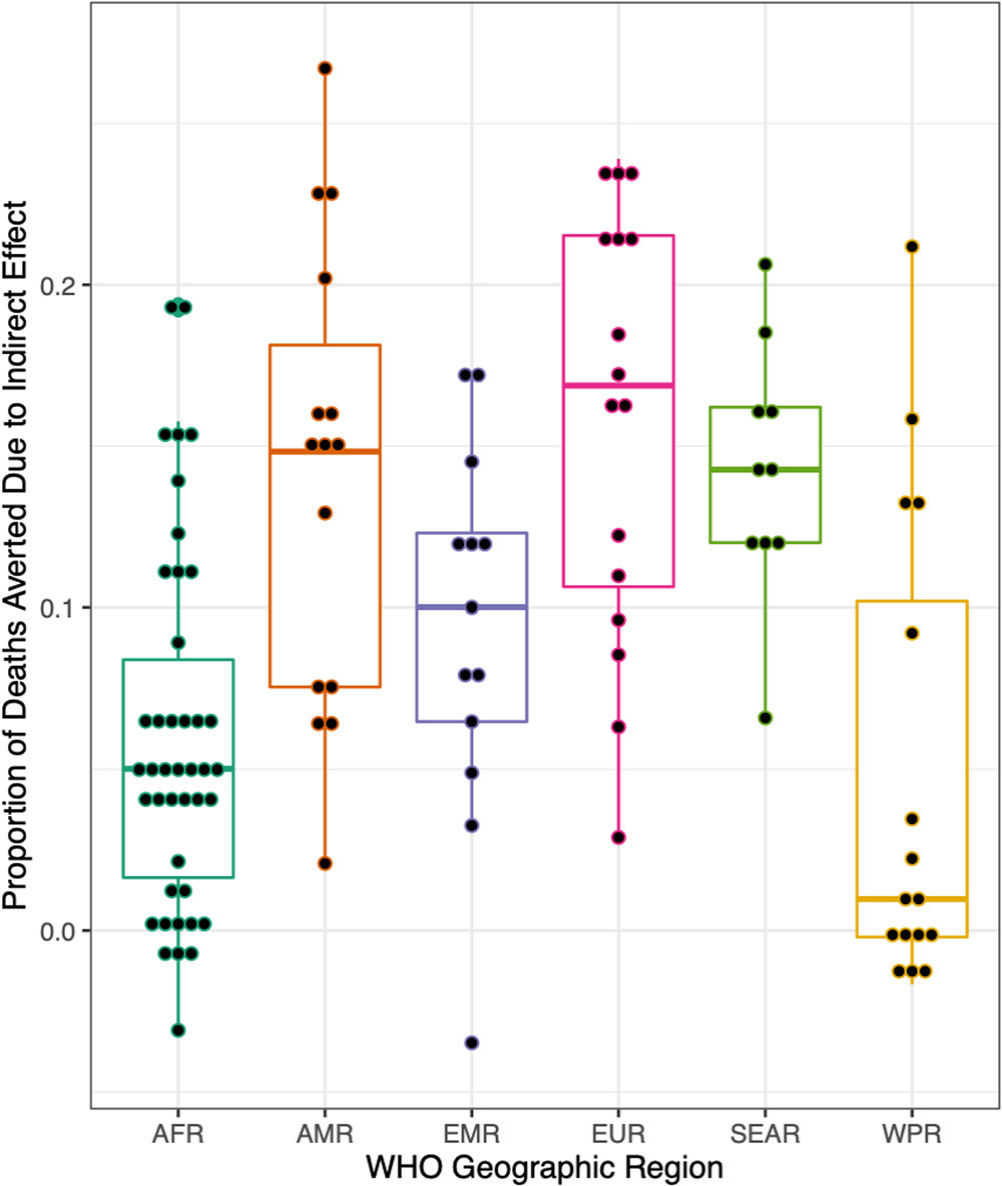
Distribution of country-level indirect effect values at 8 years post-vaccine introduction, grouped by WHO geographic region. In the boxplot below, the center line indicates the median and the lower and upper boundaries of the box represent the first and third quartile (Q1 and Q3), respectively, such that the box covers the inter-quartile range (IQR) for each region. The lower whisker is the predicted minimum value by region (Q1–1.5 x IQR) and the upper whisker is the predicted maximum value (Q3 + 1.5 x IQR) by region. Each country level estimate is shown as a point in the plot below. Dots above and below the whiskers are outliers.

In all regions, the maximum indirect effect occurred shortly after vaccine introduction, as coverage was increasing. Most countries had their maximum predicted indirect effect either during the calendar year vaccine was introduced or the following calendar year. At 8-years post-vaccine introduction, EUR countries had the highest median overall percentage of deaths averted due to indirect effects (16.9%; 2.9% to 23.9%), followed by AMR (14.8%; 2.1% to 26.7%), SEAR (14.3%; 6.6% to 20.6%), EMR (10.0%; −3.5% to 17.3%), AFR (5.0%; −3.1% to 19.4%), and WPR (0.97%; −1.7% to 21.2%) (Table 1, Fig. 2).

### Predictors of indirect effect magnitude

To estimate predictors of indirect effects, we ran linear regression models with indirect effect size (percent) for each country as the outcome. The fully adjusted model included three pre-specified covariates: birth rate, under-five mortality, and vaccine coverage (fixed effects). The country-specific birth rate was negatively associated with the indirect effect magnitude. For every 1-birth increase per 1000 people, the indirect effect decreased by 0.49% (95% CI: −0.63%, −0.36%) in Year 0, 0.64% (95% CI: −0.83%, −0.45%) in Year 5, and 0.66% (95% CI: −0.84%, −0.48%) in Year 8 (Table 2). Year-specific vaccine coverage was a significant predictor in all three models and had a positive association with indirect effect magnitude across countries. In Year 0, 5, and 8, for each 1% increase in vaccine coverage, the indirect effect increased by 0.11% (95% CI = 0.090%, 0.14%), 0.10% (95% CI = 0.037%, 0.17%) and 0.12% (95% CI = 0.054%, 0.19%), respectively (Table 2).

**Table 2:** Linear association between predicted indirect effect percent (outcome) and birth rate, under-5 mortality rate, and rotavirus vaccine coverage at 0-, 5-, and 8-years post-vaccine introduction (predictors).

	Years post-introduction of rotavirus vaccine					
	0 years		5 years		8 years	
	Unadjusted coefficient^a^ (95% C.I.)	Adjusted coefficient (95% CI)	Unadjusted coefficient (95% C.I.)	Adjusted coefficient (95% CI)	Unadjusted coefficient (95% C.I.)	Adjusted coefficient (95% CI)
**Birth rate**^b^	−0.35 (−0.45, −0.25)	−0.49 (−0.63, −0.36)	−0.51 (−0.62, −0.40)	−0.64 (−0.83, −0.45)	−0.52 (−0.63, −0.41)	−0.66 (−0.84, −0.48)
**Under-5 mortality rate**^c^	−0.08 (−0.11, −0.05)	0.02 (−0.02, 0.05)	−0.10 (−0.14, −0.06)	0.06 (0.01, 0.12)	−0.10 (−0.14, −0.07)	0.07 (0.02, 0.12)
**Vaccine Coverage (%)**	0.08 (0.04, 0.11)	0.11 (0.09, 0.14)	0.16 (0.08, 0.24)	0.10 (0.04, 0.17)	0.18 (0.10, 0.26)	0.12 (0.05, 0.19)

^a^All coefficients use the estimated indirect effect (in percent) as the outcome. For example the coefficient for vaccine coverage is the percent increase in the indirect effect per 1% increase in vaccine coverage.

^b^rate per 1000 people.

^c^rate per 1000 births.

Adjusted models include all variables in the table (birth rate, under-5 mortality rate, and vaccine coverage).

For all three models, under-5 mortality rate was negatively associated with indirect effect magnitude in the crude model but reversed direction in the fully adjusted model. The protective association in the adjusted model was statistically significant at 5- and 8-years post-vaccine introduction. For years 5 and 8, as under-5 mortality rate increased by 1 death per 1000 births, the indirect effect increased by 0.064% (95% CI = 0.010%, 0.12%) and 0.068% (95% CI = 0.016%, 0.12%), respectively (Table 2). All variance inflation factors (VIFs) were below the threshold for multicollinearity (Supplementary Table 1), and thus all pre-specified variables were included in the full model.

### Predictors of negative indirect effects

Eighteen countries had at least 1 year of predicted negative indirect effect from rotavirus vaccination during the 12-year time span (Table 3). Of these countries, 50% (*n* = 9) were from WPR, 33% (*n* = 6) from AFR, 11% (*n* = 2) from AMR, and 6% (*n* = 1) from EMR. Countries with 10 years or more of negative indirect effects tended to have higher birth rates and under-5 mortality rates than other countries and also had lower vaccine coverage (Table 3). These countries also tended to have smaller population sizes (60.6 million on average in 2022 for countries without negative indirect effects compared with 15.9 million for countries that had at least 1 year with negative indirect effects). These negative indirect effects typically occurred during time windows where the force of infection transiently increased due to predicted lapses in vaccine coverage or shortly after vaccination when the susceptible population rapidly increased. Generally, the increase in susceptible population is due to decreases in the force of infection that naturally follow vaccination, slowing the acquisition of naturally derived immunity (see Supplementary Fig. 2, more detailed discussion below). Countries without negative indirect effects tended to have smaller increases in their susceptible population after vaccine introduction. However, in the countries and years in which we predicted negative indirect effects, the value of the overall effect of the vaccine remained positive, so vaccination remained beneficial.

**Table 3:** Median birth rate, under-5 mortality rate, and mean vaccine coverage for countries stratified by the number of years for which the model predicted negative indirect effects.

	Years of negative IE		
	≥10 years	1–9 years	0 years
**Number of countries**	9	9	94
**Birth rate**^a^	36.44	25.35	25.93
**Under-5 mortality rate**^b^	92.5	28.0	41.1
**Mean vaccine coverage**^c^	50.83	76.58	80.96

^a^ rate per 1000 people.

^b^ rate per 1000 births.

^c^average of country-specific vaccine coverage from year of vaccine introduction to 12-years post-vaccine introduction.

Country-level values are shown in Supplementary Table 2.

The fully adjusted logistic regression model included birth rate, under-5 mortality rate, and mean vaccine coverage (Table 4); the outcome was an indicator variable for having at least 1 year of negative indirect effects. In this model, the odds of having at least 1 year of negative indirect effect increased by 17% with every 1 unit increase in birth rate (OR = 1.17; 95% CI = 1.04, 1.31), decreased by 4% with every 1 unit increase in under-5 mortality rate (OR = 0.96; 95% CI = 0.93, 0.99) and decreased by 5% with every 1% increase in vaccine coverage (OR = 0.95; 95% CI = 0.92, 0.98). While average vaccine coverage was lower in countries with negative indirect effects, coverage in the years negative indirect effects were observed were not always lower than average coverage for the country overall. Results were similar when average coverage at the time of indirect effects was used as the exposure of interest (Supplementary Table 3).

**Table 4:** Unadjusted and adjusted associations between birth rates, under-5 mortality rates, and mean vaccine coverage (predictors) and the occurrence of at least 1 year of negative indirect effects (outcome) from the logistic regression analysis.

	Unadjusted odds ratio (95% CI)	Adjusted odds ratio (95% CI)
**Birth rate**^a^	1.04 (0.99, 1.10)	1.17 (1.04, 1.31)
**Under-5 mortality rate**^b^	1.00 (0.99, 1.02)	0.96 (0.93, 0.99)
**Mean vaccine coverage**^c^ (%)	0.96 (0.94, 0.99)	0.95 (0.92, 0.98)

^a^rate per 1000 people.

^b^rate per 1000 births.

^c^average of country-specific vaccine coverage from year of vaccine introduction to 12-years post-vaccine introduction.

Adjusted associations include all variables in the table (birth rate, under-5 mortality rate, and mean vaccine coverage).

As with the linear models, the variables of the logistic regression models were assessed for multicollinearity, but were below the threshold of high multicollinearity (Supplementary Table 1) and thus all variables remained in the model.

## Discussion

Our modeling analysis suggests that the indirect effects of rotavirus vaccines are generally modest and vary by country-specific birth rate, under-5 mortality rate, and vaccine coverage. Lower birth rate, higher under-5 mortality rate, and higher vaccine coverage are generally associated with a stronger indirect effect and a lower likelihood of negative indirect effects. Each of these factors decrease the size of the susceptible population, which may lead to greater reductions in transmission and hence stronger indirect effects.

Our estimates indicate that the indirect effect increases the median overall effect of rotavirus vaccines in countries in all WHO geographic regions. The median indirect effect 8-years post-vaccine introduction ranged from 0.97% to 16.9% by region, which was lower than what has been seen in high-income countries^11^. Some individual countries had stronger effects, with country-specific estimates ranging from −3.5% to 26.7%. This trend has been observed in the literature, with one meta-analysis finding an average indirect effect of 25% for LMICs, which falls into the range in this study^11^. High-income countries generally have a higher direct effect of rotavirus vaccines due to biological and environmental factors that interfere with vaccine effectiveness in low-income settings^13,14^. When the direct effect is higher, lower vaccine coverage is needed to generate indirect protection^11^.

When analyzing the demographic characteristics of the countries in aggregate by geographic region across a 12-year time span, higher indirect effects were predicted for regions with lower median birth rates and under-5 mortality rates and higher vaccine coverage. This aligns with previous studies that have shown birth rates to be a determining factor in rotavirus incidence^15^. A higher birth rate will decrease the indirect effect of vaccination because as more immune-naïve infants enter the population, the number of susceptible individuals in the population increases^15^. This can lead to a higher force of infection if there is not adequate vaccine coverage, as these new births may become infected and then transmit; higher birth rates therefore diminish the indirect effect^16^. Increased vaccine coverage would act in a similar way to decreased birth rates, as it also decreases the number of susceptible people and transmission rates.

Under-5 mortality rate had a positive association with the predicted indirect effect in the fully adjusted model. Although this result is logically sound, with a higher mortality rate leading to a reduced number of susceptible individuals, it contradicts the crude associations. This discrepancy could be because the under-5 mortality rate used in the model was fixed, so decreases in this parameter due to rotavirus vaccine introduction or other public health measures were not accounted for. The crude negative association with under five mortality might also be a proxy for development and the higher background force of infection generally experienced in countries with high under-5 mortality. Controlling for birth rate and vaccine coverage in the adjusted model may have corrected bias in the crude association.

Although other dynamic rotavirus modeling studies have been published, few consider multiple countries over an extended time span, making cross-country and year comparisons difficult^17^. A strength of this analysis is that our model includes estimates for 112 countries over up to 34 years post-vaccine introduction^12^, allowing us to make longitudinal vaccine effect estimates that can be compared across countries and geographic regions. However, we note that the relationships found in this analysis pertain to the predicted effects within a model system. Real-world associations may differ based on factors not accounted for in the dynamic model.

Of the 112 countries included in this analysis, 18 had at least 1 year with an estimated negative indirect effect. The factors underlying occurrence of negative indirect effects were the same as those that predicted a lower magnitude of indirect effect; these factors tended to increase the size of the susceptible population, as described above. Most of the countries with a predicted negative indirect effect were in WPR. Countries in this region that introduced rotavirus vaccines did so relatively recently and had low initial vaccine uptake. While the date of introduction does not impact the existence of negative indirect effects, it does imply that many countries in WPR currently have a high number of children without access to rotavirus vaccine^6^. This highlights the importance of increasing access to rotavirus vaccination, particularly in regions with high birth rates.

This study has several limitations. First, our model assumes that rotavirus vaccinations are administered at exactly 2 and 4 months of age. Although this is similar to the recommended schedule for Rotarix (the most common vaccine used in low- and middle-income countries), vaccinations may be delayed due to natural infection or country-specific vaccination timelines^18,19^. Moreover, many LMICs are in the process of switching to different rotavirus vaccines (Rotasiil and Rotavac), which both require three doses^20^. Thus, the ultimate overall impacts of rotavirus vaccination and the strength of indirect effects may differ from what we have found here. Additionally, the regression model assumes that birth rates and under-5 mortality rates remain constant for each country. Year-specific birth rates are implicitly included in population size estimates used in the model, but the regression inputs do not change by year. Additionally, forecasted vaccine coverage is uncertain at the country level and actual coverage levels achieved may differ from what we have modeled.

Our simulations suggest that rotavirus vaccination provides indirect protection to unvaccinated individuals in LMICs, increasing the predicted overall impact of vaccination. The birth rate, under-5 mortality rate, and vaccine coverage may affect the strength of the indirect effect. This highlights the importance of expanding and maintaining rotavirus vaccine coverage in LMICs. Even if indirect effects are more modest in low-income settings, the overall effects of rotavirus vaccines are still substantial, and the absolute impact is larger in LMICs given the greater baseline burden.

## Methods

### Transmission model

**Fig. 3:**
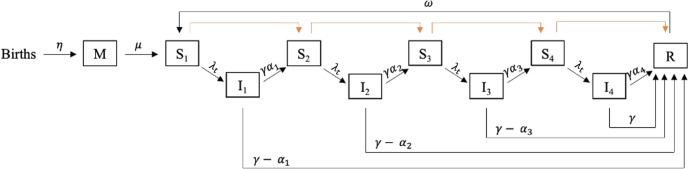
The structure of the transmission model. After birth, children have maternal immunity to the virus and enter the “M” class. When maternal immunity wanes, children become fully susceptible to infection (S1 class). After a child becomes infected (I1 class), they will either become susceptible to subsequent infection (S2 class) or fully recover and gain immunity (R class). This continues until the child reaches a fourth infection, after which they become fully recovered (R class). Infection becomes less severe with each subsequent infection. Immunity wanes and an individual will eventually become susceptible to infection again (re-enter the S1 class). The orange arrows represent the effect of vaccination. After each dose of vaccine, vaccinated individuals move to the next class of susceptibility.

This analysis utilized a published age-structured compartmental transmission model^12^ to estimate the country-specific impact of rotavirus immunization on deaths for 112 LMICs from 2000–2034 (Fig. 3), including countries from all WHO geographic regions. We generated estimates of direct effects, indirect, and overall effects of rotavirus vaccination for each country and year, comparing a default vaccine coverage scenario based on data from Gavi, the vaccine alliance, with no vaccination. Additionally, we conducted a regression analysis to identify predictors of the magnitude of indirect effects (linear regression) in the year of vaccine introduction, 5 years post-introduction, and 8 years post-introduction, and factors associated with ever having a negative indirect effect in the first 12 years after vaccine introduction (logistic regression).

### Transmission model analysis

In brief, the model accounts for maternal immunity and repeated infections, with primary infections being most likely to cause severe disease and subsequent infections having decreased likelihood of severe outcomes. Vaccination is modeled as a two-dose vaccine administered at 2- and 4-months of age (Rotarix like), with each dose conferring protection similar to one natural infection. Model development and analysis was supported by Gavi, the Vaccine Alliance, with the aim of guiding rotavirus vaccine rollout decisions globally. Using this model, we estimated rotavirus deaths in children aged 0–4 years from years 2000–2034, comparing the vaccine and no vaccine scenarios. Individual effects were estimated for 112 countries but, for simplicity, are presented in aggregate by World Health Organization geographic region along with the predicted range of effect sizes for each region. Country-specific indirect effect sizes at 8 years post-vaccine introduction are presented in Table S1. The overall effect for each country is:

$$1 - \frac{{deaths|vaccination}}{{deaths|no\,vaccination}}$$

To estimate the population direct effect of the vaccine, we ran the model with the force of infection fixed to its pre-vaccination value, which served as a counterfactual of the overall incidence if vaccine introduction did not provide indirect benefits (through reduced transmission). The force of infection is the rate at which susceptible individuals become infected with a pathogen per unit time, which generally declines after vaccine introduction^21^. The estimated country-level population direct effect is:

$$1 - \frac{{deaths|vaccination\,with\,fixed\,force\,of\,infection}}{{deaths|no\,vaccination}}$$

The estimated indirect effect was then calculated as the difference between the estimated overall effect and the estimated direct effect.

### Demographic parameters and predictors of indirect effects

Country-level socio-demographic factors were used to estimate deaths due to rotavirus and were also examined as predictors of indirect effect magnitude in the regression analysis. The median birth rate, under-5 mortality rate, and estimated vaccine coverage at 0-, 5-, and 8-years post-vaccine introduction of the countries by WHO geographic region are described in Table 5 and were used as predictors in the regression model.

**Table 5:** Median birth rate, under-5 mortality rate, rotavirus mortality rate, and vaccine coverage of countries grouped by WHO geographic region.

		WHO Geographic Region				
	AFR	AMR	EMR	EUR	SEAR	WPR
**Countries (*n*)**	42	15	13	16	10	16
**Birth rate**^a^	35.98	20.63	28.18	13.14	18.33	25.03
(Country-level range)	(20.75–47.50)	(10.82–25.63)	(18.71–42.30)	(8.66–32.12)	(10.84–29.32)	(12.31–33.56)
**Under-5 mortality rate**^b^	72.1	20.4	32.0	15.0	34.4	27.8
(Country-level range)	(24.5–156.9)	(5.5–69.0)	(12.9–136.8)	(4.6–51.4)	(9.8–52.6)	(10.7–66.7)
**Rotavirus mortality rate**^c^	38.8	5.0	21.4	0.3	7.0	4.4
(Country-level range)	(1.9–364.3)	(0.4–21.4)	(0.2–119.9)	(0.1–7.1)	(1.0–25.7)	(0.8–51.8)
**Vaccine Coverage (%)** (Country-level range)						
Year 0	31.0	38.0	12.0	10.5	0.0	0.0
	(0.0–87.0)	(0.0–82.0)	(0.0–88.0)	(0.0–99.0)	(0.0–59.7)	(0.0–85.0)
Year 5	80.5	90.0	75.0	86.6	91.7	89.6
	(53.6–98.0)	(48.0–99.0)	(49.1–98.9)	(48.0–98.8)	(77.6–98.2)	(0.0–99.0)
Year 8	82.1	90.0	87.1	91.5	88.6	89.1
	(58.0–97.1)	(53.3–98.0)	(42.0–99.0)	(56.0–98.7)	(83.2–97.4)	(0.0–99.0)

^a^rate per 1000 people.

^b^rate per 1000 births.

^c^rate per 100,000 people aged 0–4 years, 1-year pre-vaccine introduction.

The median birth rates and under-5 mortality rates for each country were assumed to be constant between years within the model. The transmission model included country- and year-specific data on age-stratified population size and vaccine coverage. The transmission model also included baseline country-specific (but time-invariant) data on the birth rate, rotavirus case-fatality ratio, and an estimated force of infection for each country based on the predicted average age of first severe infection by site. Under-five mortality was not used to parameterize the transmission model because births were set equal to deaths to avoid fluctuations in population size.

For the force of infection, we used a linear regression model to estimate the mean age in weeks of children under five with clinic visits or hospital admission for rotavirus diarrhea for each country using surveillance data from the Global Rotavirus Surveillance Network (GRSN)^22^. The final model was selected to minimize prediction error and included: under 5 mortality rate, birth rate, life expectancy, percent of population living in a rural setting, and total gross domestic product as predictors. Not all countries used in our transmission model had GRSN data for estimation, and thus for these countries the average age of first infection was predicted based on the fitted regression model. The ratio between average expectancy at birth (from 2017 estimates from United Nations Population Prospect^23^) and this estimated age of first infection was used to calculate the basic reproduction number for the model overall and the corresponding force of infection for each country^12^.

Vaccination coverage data were provided by Gavi, the Vaccine Alliance. All coverage data prior to 2020 is based on data whereas coverage between 2020 and 2034 is forecasted. The Gavi operational forecast is prepared to provide an aggregate, long-term strategic picture of the portfolio of Gavi vaccines and, as such, is highly uncertain at the country level. Based on information from other vaccines regarding the relationship between the timing of vaccine introduction and achieved vaccine coverage and using an accelerated failure time model, the Gavi forecast predicted that all countries would introduce rotavirus vaccination in 2022 if they had not done so already. Accordingly, all countries were modeled as introducing rotavirus vaccination by 2022^24^.

The analysis included countries from all WHO geographic regions, with the highest number of countries from the African region (AFR). AFR countries had the highest median birth rate (36.0 births per 1000 people) and European region (EUR) had the lowest (13.1 births per 1000 people). AFR countries also had the highest median under-5 mortality rate with 72.1 deaths per 1000 births and EUR had the lowest (15.0 deaths per 1000 births).

Estimates from the year of vaccine introduction (Year 0), 5-years post-introduction (Year 5), and 8-years post-introduction (Year 8) were used in the linear regression analysis. In the first year of introduction, Americas region (AMR) countries had the highest median vaccine coverage (38%), followed by AFR (31%), Eastern Mediterranean Region (EMR) (12%), EUR (10.5%), Western Pacific Region (WPR) (0%), and Southeast Asia Region (SEAR) (0%). SEAR and WPR had initial median coverage of 0% due to low initial uptake in these regions. All regions had a median coverage estimate of at least 75.0% by Year 5 and 82.1% by Year 8.

### Death calculation

The number of deaths was calculated by multiplying the number of severe rotavirus cases in each country per year by the case fatality ratio (CFR) for the country in each age group. Using data from the literature, the proportion of people expected to develop severe infections was estimated based on the number of infections an individual previously had and the average age of first infection in the population. The country-specific CFRs were estimated by dividing the country-specific number of expected deaths before the rotavirus vaccine became available in 2005 by the estimated number of severe cases (CFR = expected deaths / number of severe cases). The estimates for the number of expected deaths used in this calculation came from the Global Burden of Disease Study^1^.

### Regression analysis

#### Indirect effects magnitude

To identify factors driving the variation in indirect effects across countries, we analyzed predictors of the magnitude of the indirect effect using linear regression. Linear regression was deemed appropriate as the indirect effect sizes appeared normally distributed and were sufficiently below 100%, the upper bound (Supplementary Fig. 3). Each model included the country-level indirect effect size as the outcome (in percent), and separate models were run for 0, 5, and 8-years post-vaccine introduction. We assessed the impact of birth rate, under-5 mortality, and rotavirus vaccine coverage (in the year corresponding to each indirect effect estimate) on the magnitude of indirect effects and ran models with each covariate individually (unadjusted models) and with all covariates included (adjusted models). Our model did not predict biennial epidemics following vaccine introduction, so we used single year estimates. The 8-year timepoint was chosen as the final timepoint to assess because the effects were generally stable around this time.

#### Negative indirect effects

Negative indirect effects occur when the population direct effect of the vaccine is greater than the overall effect. This indicates a detrimental effect for unvaccinated individuals and vaccinated individuals who are not protected. A logistic regression model was fitted to assess the association between birth rate, under-5 mortality rate, and vaccine coverage and whether a country ever had an estimated negative indirect effect in the 12-year time span analyzed. All predictors appeared to have linear associations with the outcome, so we did not explore non-linear coefficients further. Average vaccine coverage over the 12-year period was used as the exposure of interest. As a sensitivity analysis, we also considered vaccine coverage when negative indirect effects were observed.

### Ethical considerations

This study did not require institutional review board approval because it did not include human subjects.

### Reporting summary

Further information on research design is available in the Nature Research Reporting Summary linked to this article.

## Supplementary information

Supplemental material

REPORTING SUMMARY

## Data Availability

Model output data files and input coverage estimates are proprietary of the Vaccine Impact Modelling Consortium and will not be publicly available. The authors do not have permission to share these data.

## Code availability

All code necessary to reproduce the main results of the analysis are available at: https://github.com/lopmanlab/VIMC_public. All analysis was conducted in R version 4.2.1.
