# Supplementary material for: Predicting indirect effects of rotavirus vaccination programs on rotavirus mortality among children in 112 countries
Source: NPJ Vaccines. 2023 Mar 4;8:32. doi: 10.1038/s41541-023-00632-y (PMC9985632; doi:10.1038/s41541-023-00632-y)
Supplement: Supplementary file 1 — Supplemental material [file 41541_2023_632_MOESM1_ESM.pdf]

Supplemental Information

Supplementary notes

We modeled the impact of rotavirus vaccination in 112 countries. These countries are listed below:

Afghanistan, Angola, Albania, Armenia, Azerbaijan, Burundi, Benin, Burkina Faso, Bangladesh, Bosnia and Herzegovina, Belarus, Belize, Plurinational State of Bolivia, Bhutan, Central African Republic, China, Cote d'Ivoire, Cameroon, the Democratic Republic of Congo, Congo, Colombia, Comoros, Cabo Verde, Cuba, Djibouti, Algeria, Ecuador, Egypt, Eritrea, Ethiopia, Fiji, Federated States of Micronesia, Georgia, Ghana, Guinea, Guinea-Bissau, Guatemala, Guyana, Honduras, Haiti, Indonesia, India, Islamic Republic of Iran, Iraq, Jamaica, Jordan, Kenya, Kyrgyzstan, Cambodia, Kiribati, Lao People's Democratic Republic, Liberia, Sri Lanka, Lesotho, Morocco, Republic of Moldova, Madagascar, Marshall Islands, the former Yugoslav Republic of Macedonia, Mali, Myanmar, Mongolia, Mozambique, Mauritania, Malawi, Namibia, Niger, Nigeria, Nicaragua, Nepal, Pakistan, Peru, Philippines, Papua New Guinea, Democratic People's Republic of Korea, Paraguay, State of Palestine, Rwanda, Sudan, Senegal, Solomon Islands, Sierra Leone, El Salvador, Somalia, Serbia, South Sudan, Sao Tome and Principe, Swaziland, Syrian Arab Republic, Chad, Togo, Thailand, Tajikistan, Turkmenistan, Timor-Leste, Tonga, Tunisia, Tuvalu, United Republic of Tanzania, Uganda, Ukraine, Uzbekistan, Bolivarian Republic of Venezuela, Viet Nam, Vanuatu, Samoa, Kosovo, Yemen, South Africa, Zambia, Zimbabwe

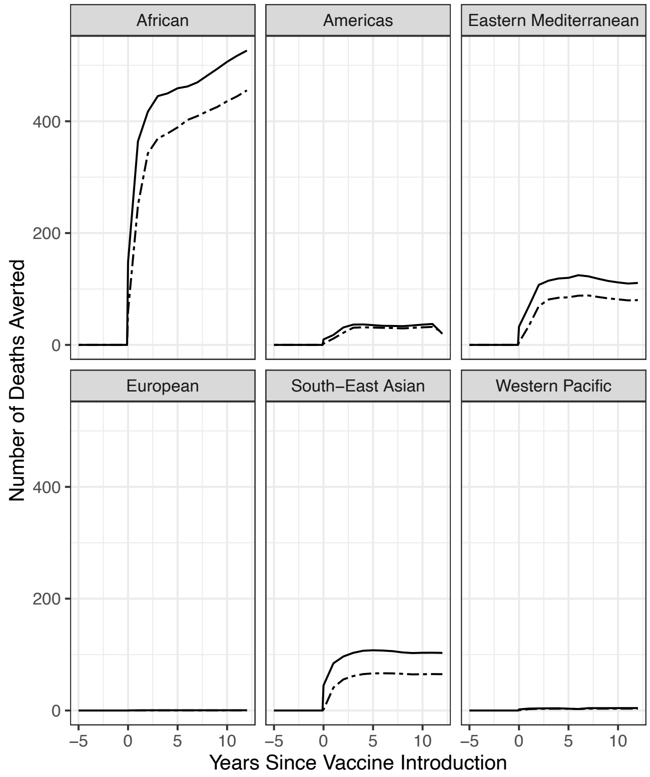

Supplementary Figure 1. Annual median number of rotavirus deaths averted across countries by region due to the population direct effect (dashed line) and overall effect (solid line) up to 12 years post vaccine introduction by WHO geographic region.

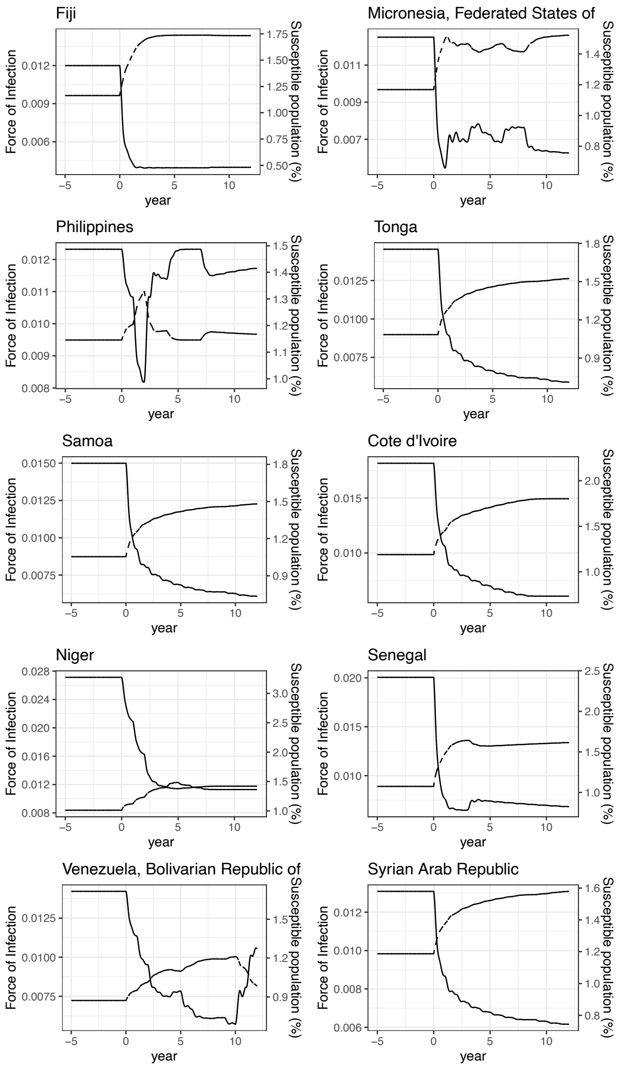

Supplementary Figure 2. Force of infection and susceptible population size for countries with negative indirect effects. Solid lines show the modeled force of infection by years since vaccine introduction and dashed lines show the proportion of the population in each country susceptible to rotavirus infection.

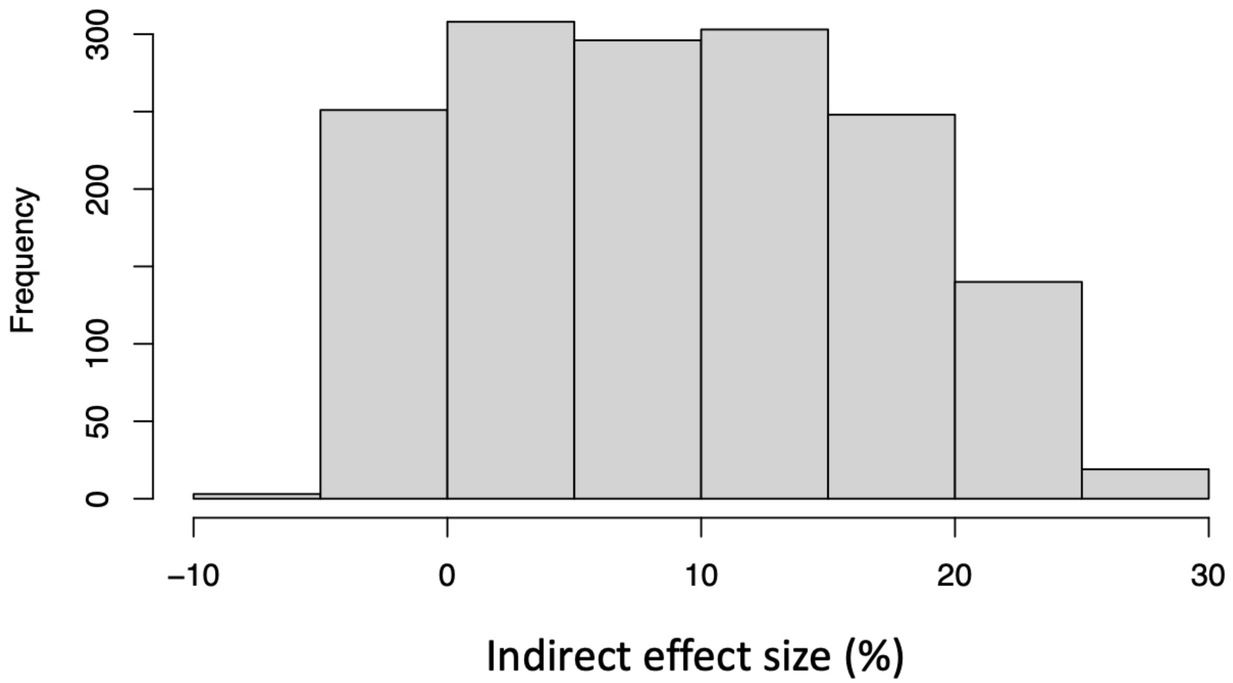

Supplementary Figure 3. Distribution of indirect effect sizes for all countries

Supplementary Table 1. Variance inflation factor (VIF) values for each parameter in the linear and logistic regression models

	Birth Rate	Under-5 Mortality Rate	Vaccine Coverage
**Linear Regression**			
Year 0	3.26	3.07	1.12
Year 5	2.95	3.24	1.22
Year 8	2.95	3.19	1.18
**Logistic Regression**	3.05	3.41	1.25

Supplementary Table 2. Indirect effect size, years with negative indirect effects, and overall predictors by country and region. Vaccine coverage is not shown due to policy restrictions from Gavi, the Vaccine Alliance.

WHO Region	Country	Year(s) Post-Vaccine Introduction w/ Negative IE Estimate	IE 8-years post introduction	Birth Rate+	Under-5 Mortality Rate++
**WPR**					
	China	N/A	21.2%	12.41	10.7
	Fiji	2-3	0.4%	21.98	22.4
	Federated States of Micronesia	1-7, 9-12	2.2%	23.21	34.7
	Cambodia	N/A	13.3%	23.67	28.7
	Kiribati	3-12	-0.6%	28.98	55.9
	Lao People's Democratic Republic	N/A	9.2%	24.71	66.7
	Marshall Islands	1,4	1.4%	23.21	36.0
	Mongolia	N/A	15.8%	25.85	22.4
	Philippines	2-6,9	0.5%	22.27	28.0
	Papua New Guinea	N/A	0%	27.99	57.3
	Solomon Islands	N/A	3.5%	33.56	28.1
	Tonga	2-8	-0.8%	25.35	16.7
	Tuvalu	8	-0.1%	25.35	27.1
	Vietnam	N/A	13.1%	17.26	21.7
	Vanuatu	2-12	-1.5%	30.81	27.5
	Samoa	2-12	-1.7%	25.43	17.5
**AFR**					
	Angola	N/A	5.2%	42.5	156.9
	Burudi	N/A	4.6%	40.9	81.7
	Benin	N/A	1.0%	37.4	99.5
	Burkina Faso	N/A	0.2%	39.4	88.6
	Central African Republic	N/A	6.6%	36.5	130.1
	Cote d'Ivoire	2-12	-0.1%	36.4	92.6
	Cameroon	N/A	4.4%	37.0	87.9
	Democratic Republic of the Congo	N/A	4.2%	42.8	98.3
	Congo	N/A	6.2%	34.5	45.0
	Comoros	N/A	6.8%	33.3	73.5
	Cabo Verde	N/A	15.8%	20.8	24.5
	Algeria	N/A	12.3%	25.5	25.5
	Eritrea	N/A	4.7%	32.2	46.5
	Ethiopia	N/A	4.9%	33.6	59.2
	Ghana	N/A	4.5%	30.7	61.6

Guinea	2-12	-1.2%	37.6	93.7
Gambia	N/A	0.5%	39.7	68.9
Guinea-Bissau	2-12	-0.2%	36.8	92.5
Kenya	N/A	13.9%	30.7	49.4
Liberia	N/A	3.6%	34.3	69.9
Lesotho	N/A	19.4%	27.8	90.2
Madagascar	N/A	5.3%	33.4	49.6
Mali	N/A	2.1%	43.2	114.7
Mozambique	N/A	11.5%	38.6	78.5
Mauritania	N/A	6.5%	35.0	84.7
Malawi	N/A	6.2%	35.8	64.0
Nambia	N/A	14.9%	29.9	45.4
Niger	2-12	-3.1%	47.5	95.5
Nigeria	N/A	1.5%	39.4	108.8
Rwanda	N/A	6.5%	32.6	41.7
Senegal	3-6	0.2%	36.3	47.2
Sierra Leone	N/A	5.3%	35.1	120.4
South Sudan	N/A	4.6%	36.1	92.6
Sao Tome and Principe	N/A	8.9%	33.0	47.3
Swaziland	N/A	19.2%	27.6	60.7
Chad	2-12	-0.8%	43.7	138.7
Togo	N/A	0.5%	34.5	78.4
United Republic of Tanzania	N/A	5.1%	37.9	48.7
Uganda	N/A	3.9%	40.7	54.6
South Africa	N/A	11.1%	21.7	40.5
Zambia	N/A	10.7%	38.0	64.0
Zimbabwe	N/A	21.5%	34.0	70.7
**AMR**				
Belize	N/A	16.1%	21.7	16.5
Bolivia	N/A	6.1%	22.8	38.4
Colombia	N/A	14.8%	15.5	15.9
Cuba	N/A	22.6%	10.8	5.5
Ecuador	N/A	7.5%	20.4	21.6
Guatemala	4	2.1%	25.6	29.1
Guyana	N/A	20.2%	20.6	39.4
Honduras	N/A	15.9%	22.4	20.4
Haiti	N/A	12.9%	25.6	69.0
Jamaica	N/A	14.6%	16.6	15.7
Nicaragua	N/A	15.5%	21.9	22.1
Peru	N/A	6.7%	18.5	16.9
Paraguay	N/A	26.7%	21.2	20.5
El Salvador	N/A	23.1%	18.7	16.8
Bolivarian Republic of Venezuela	11-12	7.6%	19.0	14.9
**EMR**				

Afghanistan	N/A	10.0%	34.8	91.1
Djibouti	N/A	11.6%	22.9	65.3
Egypt	N/A	11.9%	28.2	24.0
Islamic Republic of Iran	N/A	14.5%	19.1	15.5
Iraq	N/A	6.5%	31.1	32.0
Jordan	N/A	17.3%	24.3	17.9
Morocco	N/A	17.1%	20.3	27.6
Pakistan	N/A	3.3%	29.1	81.1
Sudan	N/A	7.7%	33.3	70.1
Somalia	N/A	4.9%	42.3	136.8
Syrian Arab Republic	2-12	-3.5%	25.0	12.9
Tunisia	N/A	12.3%	18.7	14.0
Yemen	N/A	15.7%	32.2	41.9
**EUR**				
Albania	N/A	16.0%	12.2	14.0
Armenia	N/A	11.0%	14.8	14.1
Azerbaijan	N/A	17.2%	18.5	31.7
Bosnia and Herzegovina	N/A	23.0%	8.7	5.4
Belarus	N/A	18.5%	12.2	4.6
Georgia	N/A	16.5%	14.1	11.9
Kyrgyzstan	N/A	6.3%	27.3	21.3
Republic of Moldova	N/A	23.0%	10.6	15.8
The former Yugoslav Republic of Macedonia	N/A	23.9%	11.2	5.5
State of Palestine	N/A	9.6%	31.2	21.1
Serbia	N/A	21.6%	9.6	6.7
Tajikistan	N/A	2.9%	32.1	44.8
Turkmenistan	N/A	8.5%	26.0	51.4
Ukraine	N/A	21.2%	10.3	9.0
Uzbekistan	N/A	12.2%	22.6	39.1
Kosovo	N/A	8.1%	9.6	16.4
**SEAR**				
Bangladesh	N/A	12.0%	19.2	37.6
Bhutan	N/A	12.1%	17.8	32.9
Indonesia	N/A	14.3%	19.2	27.2
India	N/A	14.3%	18.6	47.7
Sri Lanka	N/A	11.9%	16.6	9.8
Myanmar	N/A	20.6%	18.0	50.0
Nepal	N/A	18.5%	20.8	35.8
Democratic People's Republic of Korea	N/A	16.3%	13.9	24.9
Thailand	N/A	15.8%	10.8	12.3
Timor-Leste	N/A	6.6%	29.3	52.6

+ = rate per 1,000 people

++ = rate per 1,000 births

+++ = average of country-specific vaccine coverage from year of vaccine introduction to 12-years post-vaccine introduction.

Supplementary Table 3. Sensitivity analysis with coverage for countries with negative indirect effects. For models 1 and 2, the coverage exposure for countries *without* negative indirect effects is set to the average of the 12 years following vaccine introduction. For models 3 and 4, the coverage exposure for countries without negative indirect effects is set to year 1 following vaccine introduction (around the time that most countries with negative IE began to experience them).

Model	Coverage exposure	Unadjusted OR (95% CI)	Adjusted OR (95% CI)*
1	Average 12-year coverage	0.96 (0.94, 0.99)	0.95 (0.92, 0.98)
2	Average coverage in years with negative IE	0.97 (0.94, 1.00)	0.96 (0.93, 0.99)
3	Coverage in year with first negative IE	0.99 (0.98, 1.01)	0.99 (0.97, 1.00)
4	Coverage in year prior to first negative IE	1.00 (0.98, 1.01)	0.99 (0.98, 1.01)

* Adjusted models include vaccine coverage, under-five mortality, and birth rate
