# Supplementary material for: Predicting indirect effects of rotavirus vaccination programs on rotavirus mortality among children in 112 countries
Source: NPJ Vaccines. 2023 Mar 4;8:32. doi: 10.1038/s41541-023-00632-y (PMC9985632; doi:10.1038/s41541-023-00632-y)
Supplement: Supplementary file 2 — REPORTING SUMMARY [file 41541_2023_632_MOESM2_ESM.pdf]

nature portfolio

Corresponding author(s): Alicia Kraay

Last updated by author(s): Feb 8, 2023

Reporting Summary

Nature Portfolio wishes to improve the reproducibility of the work that we publish. This form provides structure for consistency and transparency in reporting. For further information on Nature Portfolio policies, see our Editorial Policies and the Editorial Policy Checklist.

Statistics

For all statistical analyses, confirm that the following items are present in the figure legend, table legend, main text, or Methods section.

n/a	Confirmed	
☒	☐	The exact sample size (*n*) for each experimental group/condition, given as a discrete number and unit of measurement
☒	☐	A statement on whether measurements were taken from distinct samples or whether the same sample was measured repeatedly
☐	☒	The statistical test(s) used AND whether they are one- or two-sided<br>*Only common tests should be described solely by name; describe more complex techniques in the Methods section.*
☐	☒	A description of all covariates tested
☐	☒	A description of any assumptions or corrections, such as tests of normality and adjustment for multiple comparisons
☐	☒	A full description of the statistical parameters including central tendency (e.g. means) or other basic estimates (e.g. regression coefficient) AND variation (e.g. standard deviation) or associated estimates of uncertainty (e.g. confidence intervals)
☐	☒	For null hypothesis testing, the test statistic (e.g. *F*, *t*, *r*) with confidence intervals, effect sizes, degrees of freedom and *P* value noted<br>*Give P values as exact values whenever suitable.*
☒	☐	For Bayesian analysis, information on the choice of priors and Markov chain Monte Carlo settings
☒	☐	For hierarchical and complex designs, identification of the appropriate level for tests and full reporting of outcomes
☐	☒	Estimates of effect sizes (e.g. Cohen's *d*, Pearson's *r*), indicating how they were calculated

Our web collection on statistics for biologists contains articles on many of the points above.

Software and code

Policy information about availability of computer code

Data collection	No software was used to collect the data
Data analysis	All data analysis was conducted in R. The analysis includes code to run the simulation model (a master script, an ODE script, and a compilation script) as well as code necessary to run the regressions on the output (linear regression and logistic regression). These files are available at https://github.com/lopmanlab/VIMC_public

For manuscripts utilizing custom algorithms or software that are central to the research but not yet described in published literature, software must be made available to editors and reviewers. We strongly encourage code deposition in a community repository (e.g. GitHub). See the Nature Portfolio guidelines for submitting code & software for further information.

Data

Policy information about availability of data

All manuscripts must include a data availability statement. This statement should provide the following information, where applicable:

- Accession codes, unique identifiers, or web links for publicly available datasets
- A description of any restrictions on data availability
- For clinical datasets or third party data, please ensure that the statement adheres to our policy

All updated model code will be available on GitHub at the time of publication. Model output data files and input coverage estimates are proprietary of the Vaccine Impact Modeling Consortium and will not be publicly available. The authors do not have permission to share these data.

Human research participants

Policy information about studies involving human research participants and Sex and Gender in Research.

Reporting on sex and gender	There were no human subjects participants
Population characteristics	This research did not involve human subjects participants.
Recruitment	This research did not involve human subjects participants.
Ethics oversight	This study did not require institutional review board approval because it did not include human subjects.

Note that full information on the approval of the study protocol must also be provided in the manuscript.

Field-specific reporting

Please select the one below that is the best fit for your research. If you are not sure, read the appropriate sections before making your selection.

☐ Life sciences ☒ Behavioural & social sciences ☐ Ecological, evolutionary & environmental sciences

For a reference copy of the document with all sections, see nature.com/documents/nr-reporting-summary-flat.pdf

Behavioural & social sciences study design

All studies must disclose on these points even when the disclosure is negative.

Study description	This is a simulation study based on a compartmental transmission model. All data used in the analysis are publicly available except for vaccine coverage estimates, which are owned by Gavi, the vaccine alliance.
Research sample	We provide estimates of vaccine impact in 112 countries. These countries were selected through our partnership with Gavi as we have vaccine coverage data from these locations.
Sampling strategy	No sample sizes apply as this is a theoretical study with compartments corresponding to the population size by age group of each country.
Data collection	All data were publicly available except for vaccine coverage, which was provided by Gavi.
Timing	The transmission model involved simulations from 2000 to 2034. All vaccine coverage data through 2022 is estimated and thereafter is forecasted.
Data exclusions	No data were excluded.
Non-participation	There were no human subjects, so there was no non-participation.
Randomization	There were no human subjects, so there was no randomization of participants.

Reporting for specific materials, systems and methods

We require information from authors about some types of materials, experimental systems and methods used in many studies. Here, indicate whether each material, system or method listed is relevant to your study. If you are not sure if a list item applies to your research, read the appropriate section before selecting a response.

Materials & experimental systems

n/a	Involved in the study	
☒	☐	Antibodies
☒	☐	Eukaryotic cell lines
☒	☐	Palaeontology and archaeology
☒	☐	Animals and other organisms
☒	☐	Clinical data
☒	☐	Dual use research of concern

Methods

n/a	Involved in the study	
☒	☐	ChIP-seq
☒	☐	Flow cytometry
☒	☐	MRI-based neuroimaging
